# Widening the spectrum of LAMA 2 congenital muscular dystrophy (MDC1A): cobblestone malformation

**DOI:** 10.1590/0004-282X-ANP-2021-0292

**Published:** 2021-12-31

**Authors:** Luiz Fernando Monte Borella, Fernanda Veloso Pereira, Paula Maria Preto Mimura, Juliana Ávila Duarte, Luciano de Lima Villarinho, Thiago Rezende, Marcondes Cavalcante de França, Fabiano Reis

**Affiliations:** 1 Universidade Estadual de Campinas, Faculdade de Ciências Médicas, Departamento de Radiologia, Campinas SP, Brazil. Universidade Estadual de Campinas Faculdade de Ciências Médicas Departamento de Radiologia Campinas SP Brazil; 2 Pontifícia Universidade Católica de São Paulo, Faculdade de Ciências Médicas e da Saúde, Departamento de Neuropediatria, Sorocaba SP, Brazil. Pontifícia Universidade Católica de São Paulo Faculdade de Ciências Médicas e da Saúde Departamento de Neuropediatria Sorocaba SP Brazil; 3 Universidade Federal do Rio Grande do Sul, Faculdade de Ciências Médicas, Departamento de Radiologia, Porto Alegre RS, Brazil. Universidade Federal do Rio Grande do Sul Faculdade de Ciências Médicas Departamento de Radiologia Porto Alegre RS Brazil; 4 The University of Mississippi Medical Center, School of Medicine, Department of Radiology, Jackson, MS, USA. The University of Mississippi Medical Center School of Medicine Department of Radiology Jackson MS USA; 5 Universidade Estadual de Campinas, Faculdade de Ciências Médicas, Departamento de Neurologia, Campinas SP, Brazil. Universidade Estadual de Campinas Faculdade de Ciências Médicas Departamento de Neurologia Campinas SP Brazil

**Figure 1.**
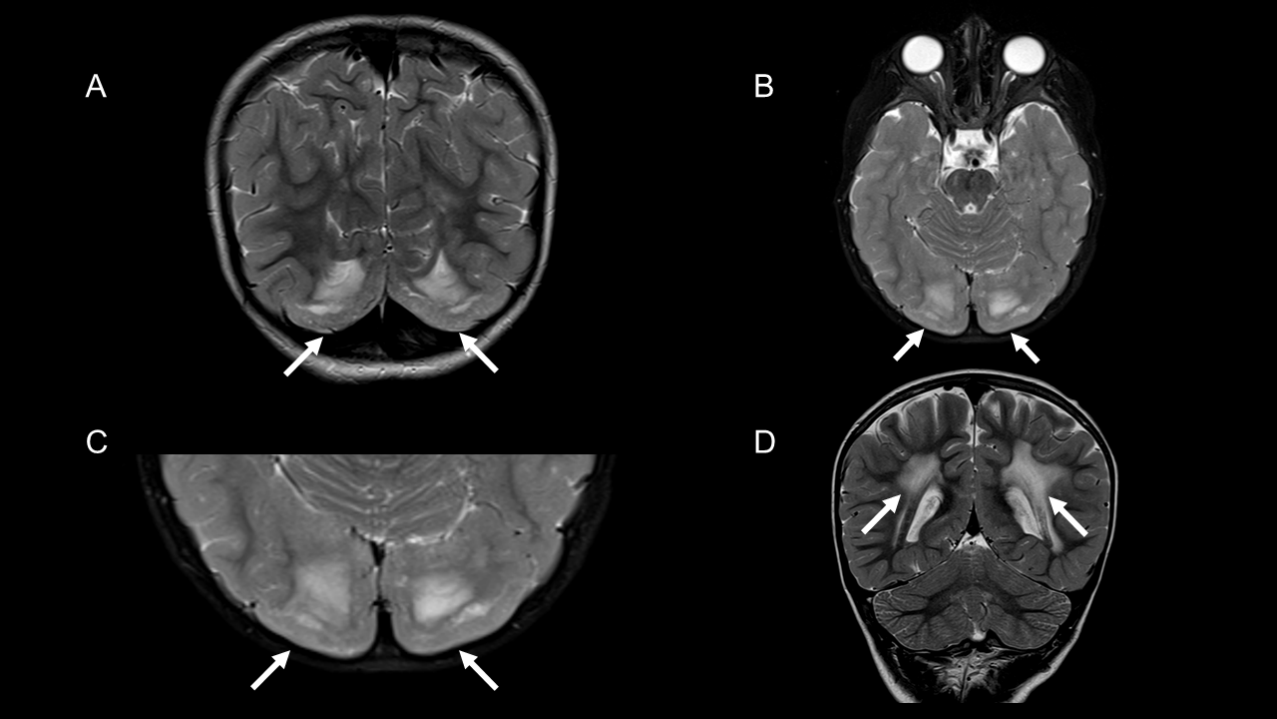
(A) Coronal T2-weighted image (T2WI) showing bilateral and symmetric type II lissencephaly or “cobblestone” lissencephaly (arrows). (B) Axial T2-weighted image (T2WI) showing bilateral and symmetric type II lissencephaly or “cobblestone” lissencephaly (arrows). (C) Axial T2-weighted image (T2WI) with a closer view of the symmetric type II lissencephaly or “cobblestone” lissencephaly (arrows), in contrast with normal cortical development. (D) Coronal T2-weighted image (T2WI) showing signal abnormalities in the periventricular white matter (arrows).

A 4-year-old boy with*LAMA2*-related congenital muscular dystrophy had two pathogenic variants (NM_000426): c.1255delA and c.2461A>C. Magnetic resonance imaging (MRI) of the brain showed signal abnormalities in supratentorial white matter (WM), which are conspicuous findings in this disease^1^. Interestingly, MRI also depicted malformations of cortical development -symmetric bilateral parieto-occipital bumpy or pebbly cortical surface (cobblestone malformation)^2^ (Figure 1).

This report expands*LAMA2*-related radiological phenotype to include not only WM abnormalities, but also predominantly posterior cerebral cortex changes.
